# 3**β**-Hydroxysterol **Δ**24-Reductase Promotes Ovarian Cancer Progression by Activating the TGF-**β**1/Smad2/3 Signaling Pathway

**DOI:** 10.32604/or.2025.065451

**Published:** 2025-09-26

**Authors:** Wenjing Liao, Liaodi Wang, Zhen Huang, Ziyu Zou, Yimin Liu, Haoyue Liu, Zhaoning Duan, Liangdan Tang

**Affiliations:** 1 Department of Gynecology and Obstetrics, The First Affiliated Hospital of Chongqing Medical University, Chongqing, 400016, China; 2 Department of Radiology, Ping An Healthcare Diagnostics Center, Wuhan, 430014, China

**Keywords:** 3β-hydroxysterol Δ24-reductase (DHCR24), metastasis, ovarian cancer (OC), proliferation, transforming growth factor beta (TGF-β1)

## Abstract

**Objectives:**

Ovarian cancer (OC) is a highly heterogeneous disease characterized by high metastatic potential and frequent recurrence. 3β-hydroxysterol Δ24-reductase (DHCR24) is closely associated with the progression of various malignant tumors, but its role in OC remains unexplored. This study is the first to systematically investigate the function of DHCR24 in OC and elucidate its mechanism in promoting OC progression, providing novel theoretical insights for targeted therapy.

**Methods:**

The expression of DHCR24 was evaluated in tissues using bioinformatics and clinical data; the impact of DHCR24 on the malignant behavior of OC was assessed through *in vivo* and *in vitro* experiments; and the mechanism by which DHCR24 functions in OC was preliminarily explored using sequencing and rescue experiments. Statistical analysis was conducted using the chi-square test, *t*-test, and one-way ANOVA.

**Results:**

Database, clinical data, and immunohistochemical (IHC) analyses demonstrated that DHCR24 is upregulated in OC and correlates with poor outcomes. *In vitro* experiments indicated that DHCR24 promotes proliferation, migration, invasion, and epithelial-mesenchymal transition in OC cells. The addition of a DHCR24 inhibitor suppressed the malignant behavior of OC cells. The nude mouse tumor formation experiment demonstrated that inhibiting DHCR24 suppresses the *in vivo* growth of OC cells. Further experiments showed that DHCR24 promotes the malignant behavior of OC cells, correlating with the regulation of the transforming growth factor beta (TGF-β) signaling pathway. All the above experiments showed statistical significance.

**Conclusion:**

DHCR24 contributes to ovarian cancer progression by upregulating the TGF-β1 pathway, highlighting its potential as a therapeutic target in ovarian cancer.

## 1 Introduction

Ovarian cancer (OC) is one of the three major gynecological cancers and has the highest mortality rate in the group [1,2]. It caused approximately 206,400 deaths globally in 2022 and affected 329,800 new patients [3]. For example, epithelial OC (EOC) accounts for the largest proportion of OC cases [4–6]. Overall, treating OC is challenging, necessitating studies into the molecular mechanisms of the disease and its key signaling pathways. This research can lead to the development of improved diagnostic tools and innovative therapies to enhance the survival outcomes of patients with OC.

3β-hydroxysterol Δ24-reductase (DHCR24), also known as seladin-1, is primarily localized on the endoplasmic reticulum membrane [7,8]. It is a single-pass transmembrane protein with multiple transmembrane domains in the cytoplasm [9]. As the key enzyme in the final step of cholesterol biosynthesis, DHCR24 catalyzes the conversion of desmosterol to cholesterol. Beyond its role in cholesterol metabolism, it extensively participates in regulating various cellular physiological processes, including neuroprotection, antioxidation, and apoptosis inhibition [10,11]. Current studies demonstrate that DHCR24 exerts antioxidant function by scavenging reactive oxygen species (ROS) and can influence tumorigenesis by responding to and regulating intracellular ROS [8,12]. In addition to its associations with Alzheimer’s disease [13], cardiovascular diseases [14,15], metabolic disorders [16], and hepatitis C virus [17,18], this enzyme is also closely linked to adverse clinical outcomes in multiple malignancies [19–21]. During cancer development, DHCR24 has been demonstrated to be aberrantly expressed in various cancers, including metastatic melanoma, bladder cancer, and salivary adenoid cystic carcinoma [22–24], although its specific mechanisms in OC remain to be fully elucidated.

Transforming growth factor beta (TGF-β) is one of the growth factors that constitute the TGF-β superfamily, with TGF-β1 being the most frequently expressed and widely studied homotype [25,26]. It promotes tumor progression through various mechanisms, including enhancing angiogenesis and inhibiting the anti-tumor function of the immune system [27,28]. Additionally, TGF-β is a key inducer of the epithelial-mesenchymal transition (EMT) process [29,30]. Although the role of DHCR24 in cholesterol metabolism and apoptosis regulation has been extensively studied, whether it influences OC cell malignant behaviors through specific signaling pathways remains unclear. As the most lethal malignancy of the female reproductive system, OC progression is closely associated with abnormal activation of the TGF-β signaling pathway. Based on DHCR24’s tumor-promoting effects in various cancers, we hypothesized that DHCR24 might promote OC malignant progression by regulating the TGF-β pathway. This study provides the first systematic evaluation of DHCR24’s role in OC and investigates its mechanisms in promoting OC progression, offering novel theoretical insights for targeted therapy and further enriching the understanding of its oncogenic functions.

## 2 Materials and Methods

### 2.1 Database Analysis

Gene expression analysis of DHCR24 in OC tissues versus normal ovarian epithelial tissues was performed using the Gene Expression Profiling Interactive Analysis 2 (GEPIA 2) (http://gepia.cancer-pku.cn), which integrates data from the Cancer Genome Atlas (TCGA) database. The analysis included 426 tumor samples and 88 normal control samples. The “Expression DIY” module in GEPIA 2 was employed for comparative analysis, with a statistical significance threshold set at *p* < 0.05. Prognostic analysis of DHCR24 expression was performed using the Kaplan-Meier Plotter (https://kmplot.com/analysis/, accessed on 14 May 2025) online platform with progression-free survival (PFS) as the primary endpoint. Patients were stratified into high- and low-expression groups based on the optimal expression cutoff value. Intergroup differences were assessed by log-rank test with a significance threshold of *p* < 0.05.

### 2.2 Tissues

In this study, paraffin sections of 70 cases of OC were examined. In addition, fresh human normal ovarian and OC tissue specimens (16 each) were collected and stored in liquid nitrogen for follow-up experiments. Patient details can be found in Table S1.

This study was approved by the Ethics Committee of the First Affiliated Hospital of Chongqing Medical University (approval number: 2024-006-02). Informed consent has been obtained from all patients.

### 2.3 Cell Culture

Normal ovarian epithelial cells IOSE-80 (RRID: CVCL_5546) were purchased from Zhong Qiao Xin Zhou Biotechnology Co., Ltd. (Shanghai, China). OC cells [SKOV3 (RRID:CVCL_0532), CAOV3 (RRID:CVCL_0201), ES2 (RRID: CVCL_3509), and OVCAR3 (RRID:CVCL_0465)]. OC cells A2780 (RRID: CVCL_0134) were purchased from Servicebio Co., Ltd. (Wuhan, China). All cell lines were authenticated by STR profiling and confirmed to be free of mycoplasma contamination. All cells were cultured in McCoy’s 5A medium (Procell, Wuhan, China) at 37°C in a 5% CO_2_ incubator.

### 2.4 RNA Extraction and Quantitative PCR (qPCR)

Total RNA was extracted from the aforementioned tissues and cells using Trizol (Cat. No. 9109, TaKaRa, Tokyo, Japan). qPCR was performed by TB Green Premix Ex Taq (Cat. No. RR820A, TaKaRa) [31,32]. Gene expression differences were calculated using the 2^−ΔΔCt^ method, with intergroup comparisons performed by *t*-tests or one-way ANOVA. Primer sequences are shown in Table S2.

### 2.5 Western Blot (WB)

Proteins (25 μg) were separated by 8% and 10% sodium dodecyl-sulfate polyacrylamide gel electrophoresis. The proteins were transferred onto a polyvinylidene difluoride membrane via electroblotting. The membrane was blocked with skim milk for 2 h, followed by incubation with primary antibodies [diluted in antibody dilution buffer (Cat. No. P0256, Beyotime, Shanghai, China)] at 4°C overnight, using GAPDH or β-Actin as controls [with the following dilution ratios: DHCR24 1:1000 (#2033, CST, Danvers, MA, USA), E-cadherin 1:1000 (#3195, CST, USA), N-cadherin 1:1000 (#13116, CST, USA), Vimentin 1:1000 (#5741, CST, USA), Bax 1:1000 (#5023, CST, USA), Bcl2 1:1000 (#3498, CST, USA), TGF-β1 1:1000 (ab215715, Abcam, Cambridge, UK), Smad2/3 1:1000 (#8685, CST, USA), Phospho-Smad2 1:1000 (#3108, CST, USA), Phospho-Smad3 1:2000 (ab52903, Abcam, UK), GAPDH 1:5000 (380626, Zen-BioScience, Chengdu, China), β-Actin 1:5000 (380624, Zen-BioScience, China)]. The next day, secondary antibody incubation (diluted in Tris-Buffered Saline with Tween-20) was performed at room temperature for 1 h. Finally, an ultrasensitive chemiluminescence kit (Cat. No. BL520A, Biosharp, Anhui, China) was used to visualize the proteins. Antibody data are presented in Table S3. All experiments were independently repeated three times.

### 2.6 Lentiviral Infection

The cells (SKOV3, CAOV3, ES2, and A2780) were seeded (1 × 10^5^ cells/well) in a 12-well plate one day in advance to achieve approximately 30% confluence by the second day. SKOV3 and CAOV3 cells were infected with a lentivirus-mediated DHCR24 low-expression vector (Hanbio, Shanghai, China). Resistant cells were screened using puromycin (2 µg/mL), resulting in stable DHCR24 low-expression cell lines SKOV3/sh1, sh2, sh3, and their control cell lines SKOV3/shNC, alongside CAOV3/sh1, sh2, sh3, and their control cell lines CAOV3/shNC. In addition, ES2 and A2780 cells were infected with a lentivirus-mediated DHCR24 overexpression vector (Hanbio). Stable DHCR24 high-expression cell line ES2/DHCR24 and its control cell line ES2/Vector, and A2780/DHCR24 and its control cell line A2780/Vector were constructed. DHCR24 interference sequences are shown in Table S4.

### 2.7 Cell Counting Kit-8 (CCK-8) Assay

Cells (3 × 10^3^ cells/well) were evenly seeded in a 96-well plate, with five replicate wells per group, and blank control wells (containing only medium + CCK-8 reagent) were also set up. Five plates were prepared simultaneously for each group (labeled as D0–D4). After 4−6 h, cell adherence was checked, and the medium was replaced with a fresh medium containing 10% CCK-8 solution (Cat. No. K1018, APExBIO, Houston, TX, USA). The plates were then returned to the incubator for further culturing. After 2 h, the D0 plate was removed, and absorbance at 450 nm was measured under light-protected conditions using a microplate reader (Infinite 200 PRO, Tecan, Switzerland), recorded as the D0 absorbance value. The same procedure was repeated at the same time for D1–D4. First, measure the raw absorbance, then subtract the blank control to calculate the corrected absorbance: Corrected absorbance = Sample well absorbance − Blank well absorbance.

### 2.8 Colony Formation Assay

Cells (SKOV3, CAOV3, ES2 and A2780) were evenly seeded (4 × 10^2^ cells/well) and cultured. Samples were collected once colonies visible to the naked eye had formed (approximately 2 weeks of culturing). Photographs were captured and the colony numbers were quantified using Fiji software (version 2.3.0).

### 2.9 Transwell Migration and Invasion Assays

For the migration assay, SKOV3 and ES2 cells (2 × 10^4^ cells/well) and CAOV3 and A2780 cells (6 × 10^4^ cells/well) were inoculated into Transwell inserts (BIOFIL, Guangzhou, China) containing fetal bovine serum-free medium, with the complete medium in the lower chamber. For the invasion assay, 80 μL of Matrigel (Cat. No. 082704, Mogengel-Bio, Xiamen, China) diluted at a 1:8 ratio was first added to the upper chamber and incubated for 3 h to allow the gel to solidify. The cells were then seeded (same as the migration assay). The upper and lower chambers were supplemented with the same medium as previously described. Samples were collected after 24 or 96 h of incubation. Specifically, after 24 h, samples were collected from SKOV3 and ES2 cells, whereas after 96 h, samples were collected from CAOV3 and A2780 cells. Samples were first fixed with 4% paraformaldehyde solution (Cat. No. P1110, Solarbio, Beijing, China) for 15 min, then stained with crystal violet solution (Cat. No. C0121, Beyotime, Shanghai, China) for 9–10 min, washed with PBS, and subsequently photographed.

### 2.10 Wound Healing Assay

Cells (SKOV3 and CAOV3) were seeded (5 × 10^5^ cells/well) in a 6-well plate and cultured for approximately 3−4 days. When the confluence reached over 90%, a 200 µL pipette tip was used to create linear scratches perpendicular to the bottom of the plate. A photomicrograph of the scratched area was taken, marking the time as 0 h. The culture was continued, and images of the scratch area were captured at appropriate time points (after 24 h for SKOV3 cells; after 72 h for CAOV3 cells) using a microscope (CKX53, OLYMPUS, Tokyo, Japan).

### 2.11 Flow Cytometry Assay

Cells (SKOV3, CAOV3, ES2, and A2780) were seeded (5 × 10^5^ cells/well) and sampled as the cells grew logarithmically. Binding buffer, annexin V-FITC, and propidium iodide (Cat. No. E-CK-A211, Elabscience, Wuhan, China) were then slowly added. Incubate the cells for 15 min in the dark before analysis. The flow cytometer used in this experiment was the CYTOFLEX (CytoFLEX S, Beckman Coulter, Brea, CA, USA).

### 2.12 Subcutaneous Tumor Model in Mice

Twenty-two BALB/c nude mice were purchased from Changzhou Cavins Laboratory Animal Co., Ltd. (Changzhou, China). The mice were housed in an AAALAC-accredited facility with controlled temperature (23 ± 1°C), humidity (50 ± 10%), and a 12-h light/dark cycle, with a 1-week acclimation period. A total of 10 mice were randomly divided into 2 groups (5 mice per group) and subcutaneously injected with OC cells (SKOV3/shNC and SKOV3/sh1, 5 × 10^6^ cells per mouse). Separately, 12 mice were randomly divided into 2 groups (6 mice per group) and injected with OC cells (A2780/DHCR24 and A2780/Vector, 5 × 10^6^ cells per mouse). Mice were maintained for 30–40 days (15 July to 25 August 2024). When the subcutaneous tumor diameter reached 1.0 cm, the mice were euthanized with pentobarbital sodium, and solid tumors were surgically extracted and weighed. The tumor tissue was fixed with a tissue-fixative solution for immunohistochemistry (IHC).

Animal experiments were approved by the Institutional Animal Care and Use Committee of Chongqing Medical University (approval number: IACUC-CQMU-2024-0705). The study was reported in accordance with ARRIVE guidelines.

### 2.13 Immunohistochemistry (IHC)

The tissue sections were baked at 60°C. Sodium citrate solution was used for antigen repair. Subsequently, the tissue sections were subjected to a blocking solution and incubated with the primary antibody. A diaminobenzidine (20x) chromogenic solution (Cat. No. ZLI-9018, ZSGB-BIO, Beijing, China) was applied. Nuclei were stained with hematoxylin (Cat. No. G1004, Servicebio, Wuhan, China). Information regarding the antibodies that were used is provided in Table S5.

### 2.14 RNA Sequencing

Transcriptome sequencing was conducted by Seqhealth Technology Co., Ltd. (Wuhan, China) using a NovaSeq 6000 sequencer (Illumina, San Diego, CA, USA). Differentially expressed genes between groups were identified using the ‘edgeR’ package (version 3.42.0) in R 4.3.0 (The R Foundation for Statistical Computing, Vienna, Austria), with screening criteria of |logFC| > 1 and adjusted *p*-value < 0.05. The results were visualized in a volcano plot using the ggplot2 R package (version 3.4.0). Subsequently, Kyoto Encyclopedia of Genes and Genomes (KEGG) pathway enrichment analysis and Gene Set Enrichment Analysis (GSEA) were conducted using the “ClusterProfiler” R package (version 4.8.0), with a significance threshold of *p* < 0.05 [33,34].

### 2.15 Rescue Experiment

SKOV3 cells were treated with TGF-β1 (Cat. No. HY-P7118, MCE, Middlesex, NJ, USA), while A2780 cells were treated with the TGF-β1 pathway inhibitor SB431542 (Cat. No. S1067, Selleck Chemicals, Houston, TX, USA). SKOV3 and ES2 cells were treated with the DHCR24 inhibitor SH-42 (Cat. No. HY-143228, MCE, NJ, USA). Functional assays were performed after 48 h, and total cellular proteins were extracted for Western blot at 72 h. Relevant experimental details can be found in the corresponding methods section. SB431542 and SH-42 were dissolved in dimethyl sulfoxide (DMSO) to a concentration of 10 μM, and TGF-β1 was dissolved in PBS (pH 7.2–7.4) containing bovine serum albumin (BSA) to a concentration of 10 ng/mL.

### 2.16 Statistical Analysis

Statistical analysis and graph plotting were performed using SPSS 25.0 and GraphPad Prism 8.0 software, while image processing was conducted with Fiji software. Continuous variables are expressed as mean ± standard deviation (Mean ± SD), with comparisons between two groups analyzed by *t*-test and multi-group comparisons by one-way ANOVA. Categorical variables were described as frequency (percentage) and compared using the chi-square test. *p* < 0.05 was considered statistically significant (*p < 0.05, **p < 0.01, ***p < 0.001), with corrections for multiple comparisons applied.

## 3 Results

### 3.1 DHCR24 Expression Is Upregulated in OC and Is Linked to Adverse Prognosis

**Figure 1:**
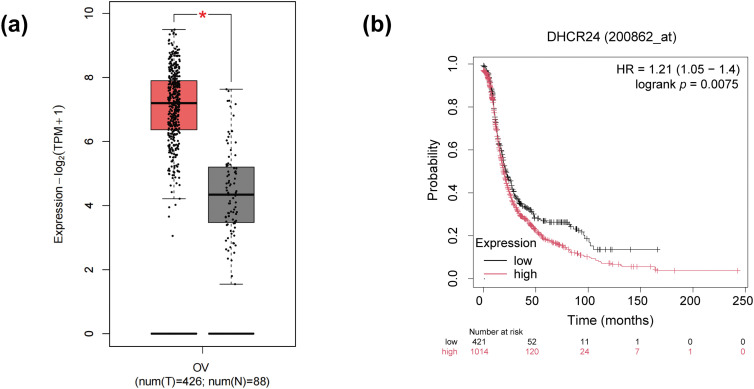

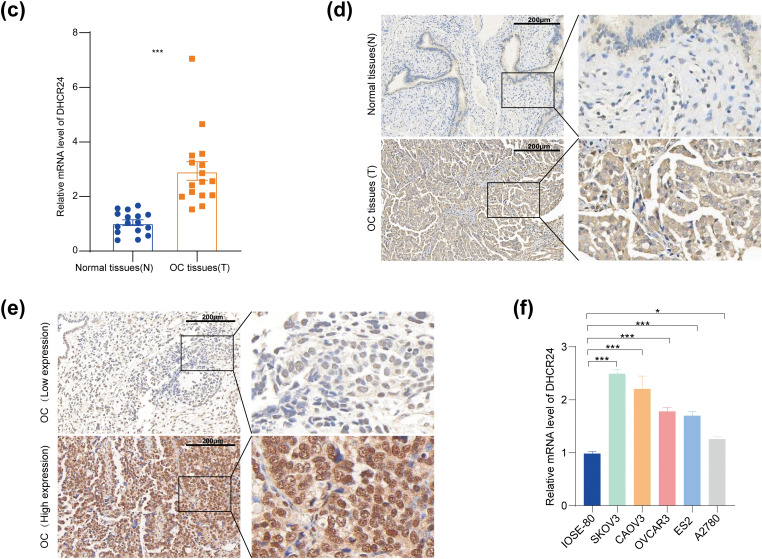
DHCR24 overexpression is associated with OC prognosis. (**a**) DHCR24 expression in normal ovarian (N) and OC (T) tissues from the GEPIA database; (**b**) Kaplan-Meier curve showing the relationship between DHCR24 expression and PFS in patients with OC; (**c**) qPCR results showing DHCR24 mRNA expression in normal ovarian (N) and OC (T) tissues (*n* = 16); (**d**) IHC results showing DHCR24 protein levels in normal ovarian (N) and OC (T) tissues; (**e**) IHC was performed to detect the expression of TGF-β1 in OC tissues; (**f**) qPCR results for DHCR24 mRNA expression in normal ovarian epithelial cells and OC cell lines. The experiment was independently repeated three times. Note: GEPIA, Gene Expression Profiling Interactive Analysis; DHCR24, 3β-hydroxysterol Δ24-reductase; KM, Kaplan–Meier; PFS, Progression-Free Survival; qPCR, quantitative PCR; mRNA, messenger ribonucleic acid; IHC, immunohistochemistry. *p < 0.05, ***p < 0.001

The GEPIA database indicated that DHCR24 in the OC tissues was higher than that in the normal tissues (Fig. 1a). Kaplan-Meier database showed shorter progression-free survival (PFS) in patients with high DHCR24 expression (Fig. 1b). Subsequently, using qPCR and IHC, we confirmed significant upregulation of DHCR24 expression in the OC tissues (*p* < 0.001) (Fig. 1c,d). To compare the relationship between DHCR24 and clinicopathological parameters of OC, we used IHC to detect the expression of DHCR24 in 70 cases of OC. Additionally, we examined the expression of TGF-β1 in OC tissues using IHC. The results showed that TGF-β1 expression was significantly higher in the DHCR24 high-expression group than in the DHCR24 low-expression group, suggesting a potential positive correlation between TGF-β1 expression and DHCR24 expression (Fig. 1e). By analyzing the pathological data of patients, we found that the level of DHCR24 was significantly related to the International Federation of Gynecology and Obstetrics (FIGO) stage (*p* = 0.015) and ascites condition (*p* = 0.027) of patients with OC. Patient data are listed in Table S1. The qPCR results suggested increased DHCR24 expression in OC cells (All *p* < 0.05) (Fig. 1f). Collectively, these findings suggested a significant increase in DHCR24 expression in OC, which is linked to undesirable outcomes for OC.

### 3.2 Construction of an In Vitro Model of Differentially Expressed DHCR24

**Figure 2:**
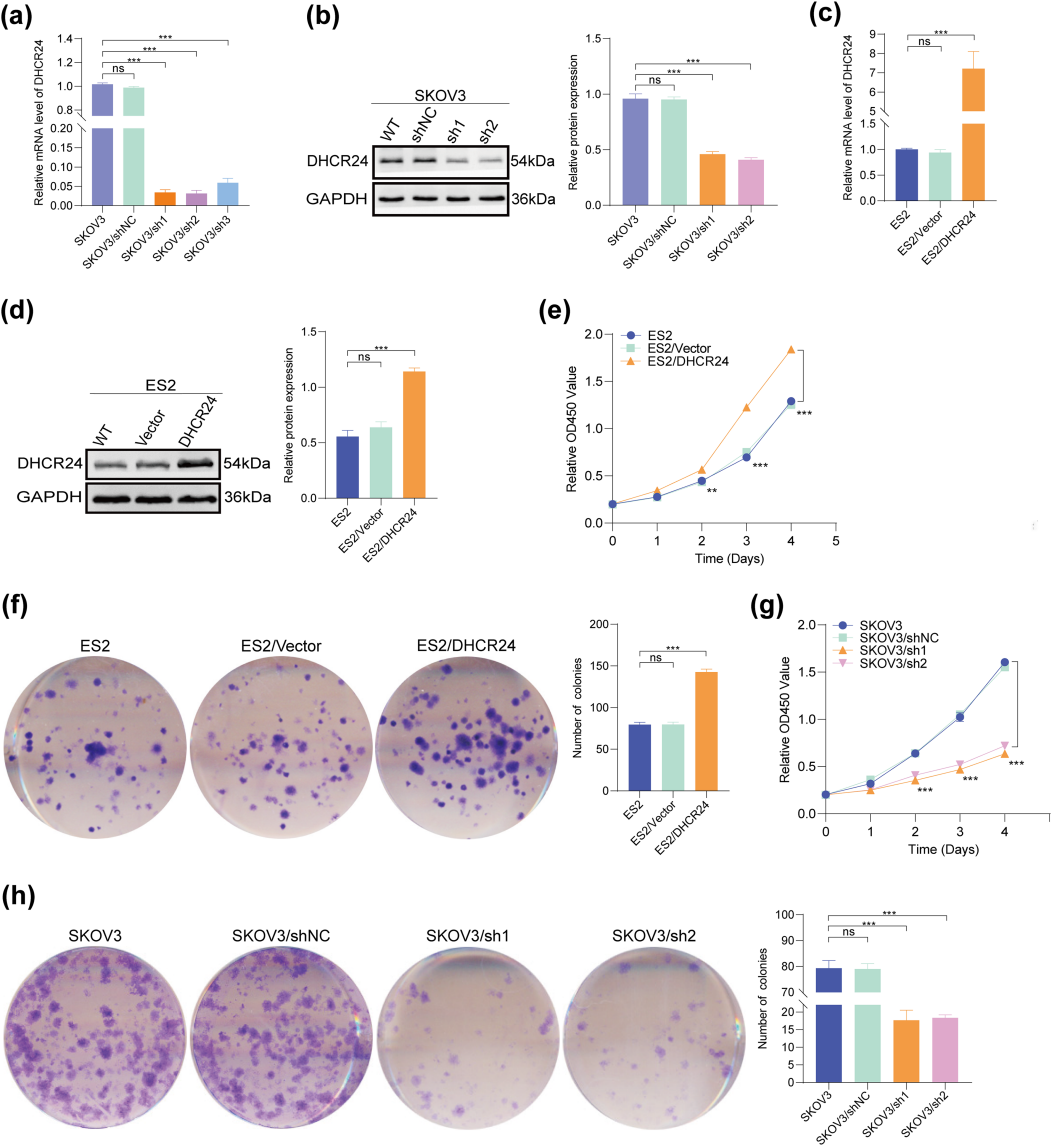
DHCR24 promotes OC cell proliferation. (**a**) Transfection efficiency of DHCR24-shRNA in SKOV3 cells, detected using qPCR; (**b**) Left: WB results verified the establishment of an *in vitro* model showing stable and low expression of DHCR24. Right: Corresponding quantitative analysis results; (**c**) Transfection efficiency of overexpressed DHCR24 in ES2 cells, detected using qPCR; (**d**) Left: Establishment of an *in vitro* model showing stable overexpression of DHCR24, verified using WB. Right: Corresponding quantitative analysis results; (**e**) CCK-8 test showing that DHCR24 overexpression promotes OC cell proliferation (3 × 10^3^ cells/well); (**f**) Left: Colony formation assay showing that overexpression of DHCR24 promotes OC cell proliferation. Right: Corresponding quantitative analysis results (4 × 10^2^ cells/well); (**g**) CCK-8 test showing that DHCR24 knockdown inhibits OC cell proliferation (3 × 10^3^ cells/well); (**h**) Left: Colony formation assay showing that DHCR24 knockdown inhibits the proliferation of OC cells. Right: Corresponding quantitative analysis results (4 × 10^2^ cells/well). All experiments were independently repeated three times. Note: DHCR24, 3β-hydroxysterol Δ24-reductase; OC, ovarian cancer; qPCR, quantitative PCR; WB, western blot; CCK-8, cell counting kit-8. ns, no significance; **p < 0.01, ***p < 0.001

SKOV3 and CAOV3 cells were used to establish an *in vitro* model with stable and low expression of DHCR24. A stable model of DHCR24 overexpression was established using ES2 and A2780 cells. Results of qPCR and WB confirmed that DHCR24 expression decreased in the cells of the DHCR24 low-expression group (SKOV3/sh1, sh2, sh3) (Fig. 2a,b) but increased in the cells of the DHCR24 high-expression group (ES2/DHCR24) (All *p* < 0.001) (Fig. 2c,d). These validations were also performed in the other two cell lines (Fig. S1).

### 3.3 DHCR24 Promotes OC Cell Proliferation

CCK-8 and clone formation experiments confirmed that DHCR24 overexpression increased proliferation and colony formation in ES2 cells (Fig. 2e,f), whereas decreased DHCR24 expression had reverse effects in SKOV3 cells (All *p* < 0.01) (Fig. 2g,h). The same experiments were performed in the other two cell lines, yielding consistent results (Fig. S1).

### 3.4 DHCR24 Inhibits Apoptosis in OC Cells

**Figure 3:**
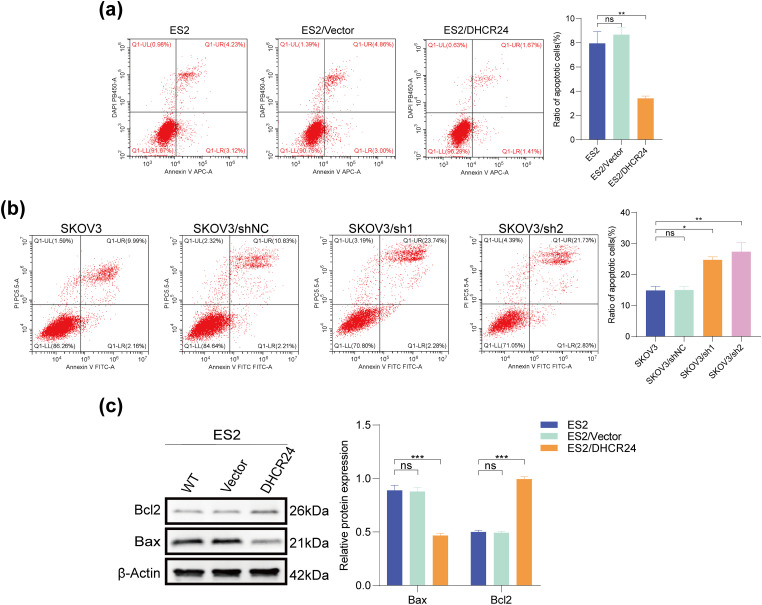

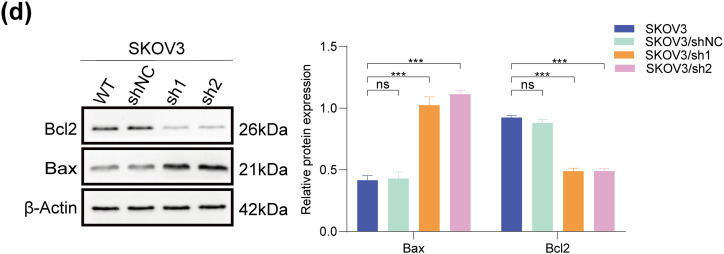
DHCR24 inhibits apoptosis of OC cells. (**a**) Left: Apoptosis of OC cells before and after DHCR24 overexpression, detected using flow cytometry. Right: Corresponding quantitative analysis results; (**b**) Left: Apoptosis of OC cells before and after DHCR24 knockdown, detected using flow cytometry. Right: Corresponding quantitative analysis results; (**c**) Left: Changes in apoptosis-related proteins Bax and Bcl2 before and after overexpression of DHCR24, detected using WB. Right: Corresponding quantitative analysis results; (**d**) Left: Changes in apoptosis-related proteins Bax and Bcl2 before and after DHCR24 knockdown, detected using WB. Right: Corresponding quantitative analysis results. All experiments were independently repeated three times. Note: DHCR24, 3β-hydroxysterol Δ24-reductase; OC, ovarian cancer; WB, western blot. ns, no significance; *p < 0.05, **p < 0.01, ***p < 0.001

Flow cytometry results showed that DHCR24 overexpression reduced apoptosis in OC cells (Fig. 3a), whereas decreased DHCR24 expression had reverse effects (Fig. 3b). In addition, WB suggested increased Bcl2 expression but decreased Bax expression in the high DHCR24 expression group (Fig. 3c), and reverse results were noted in the low DHCR24 expression group (All *p* < 0.05) (Fig. 3d). When the same experiments were performed in the other two cell lines, the conclusions were consistent (Fig. S2).

### 3.5 DHCR24 Promotes Migration and Invasion of OC Cells

**Figure 4:**
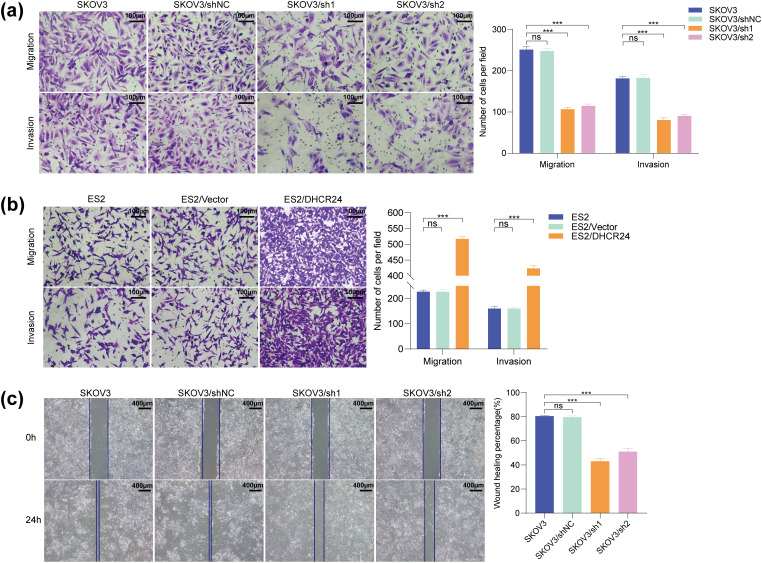

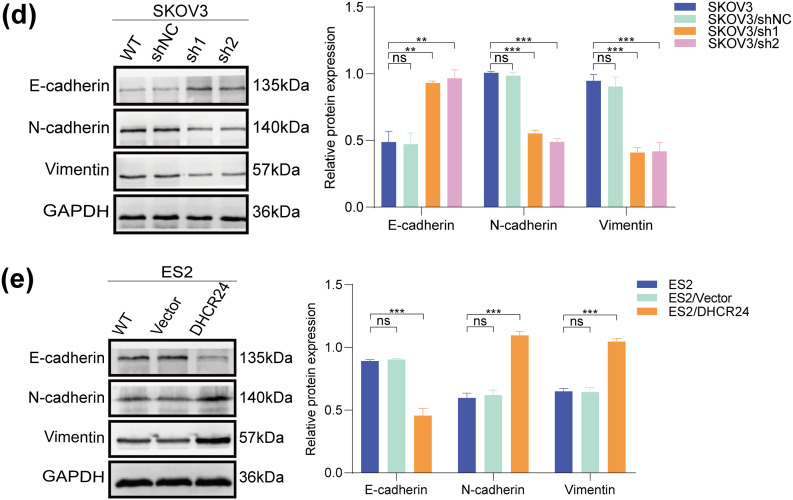
DHCR24 promotes migration, invasion, and EMT of OC cells. (**a**) Left: Transwell experiment results showing that DHCR24 knockdown inhibits OC cell migration and invasion. Right: Corresponding quantitative analysis results (SKOV3: 2 × 10^4^ cells/well); (**b**) Left: Transwell experiment results showing that DHCR24 overexpression promotes migration and invasion of OC cells. Right: Corresponding quantitative analysis results (ES2: 2 × 10^4^ cells/well); (**c**) Left: Wound healing experiment results showing that DHCR24 knockdown inhibits the migration of OC cells. Right: Corresponding quantitative analysis results; (**d**) Left: Changes in EMT-related proteins in OC cells before and after DHCR24 knockdown, detected using WB. Right: Corresponding quantitative analysis results; (**e**) Left: Changes in EMT-related proteins in OC cells before and after DHCR24 overexpression, detected using WB. Right: Corresponding quantitative analysis results. All experiments were independently repeated three times. Note: DHCR24, 3β-hydroxysterol Δ24-reductase; OC, ovarian cancer; EMT, Epithelial-mesenchymal transition; WB, western blot. ns, no significance; **p < 0.01, ***p < 0.001

Transwell experiment indicated that the migration and invasion of OC cells decreased after DHCR24 knockdown (Fig. 4a) but were enhanced in the DHRC24 overexpression group (Fig. 4b). In addition, the wound healing experiment showed that the wound healing rates of OC cells decreased in the knockdown groups (All *p* < 0.01) (Fig. 4c). The Transwell assay in CAOV3 and A2780 cells yielded results consistent with the above findings (Fig. S3).

### 3.6 DHCR24 Promotes EMT in OC Cells

To demonstrate the effect of DHCR24 on EMT, we performed WB and found that the levels of E-cadherin in the DHCR24 low-expression group (SKOV3/sh1, sh2) were higher than that in the control group (SKOV3/shNC and SKOV3), whereas the levels of N-cadherin and Vimentin decreased (Fig. 4d). In ES2 cells, compared with the control group (ES2/Vector and ES2), E-cadherin expression in the DHCR24 overexpression group (ES2/DHCR24) decreased, whereas N-cadherin and Vimentin expression increased (All *p* < 0.001) (Fig. 4e). When the same experiments were performed in the other two cell lines, the conclusions were consistent (Fig. S3). Taken together, these results suggested that DHCR24 promotes EMT in OC cells.

### 3.7 DHCR24 Promotes OC Cell Proliferation In Vivo

**Figure 5:**
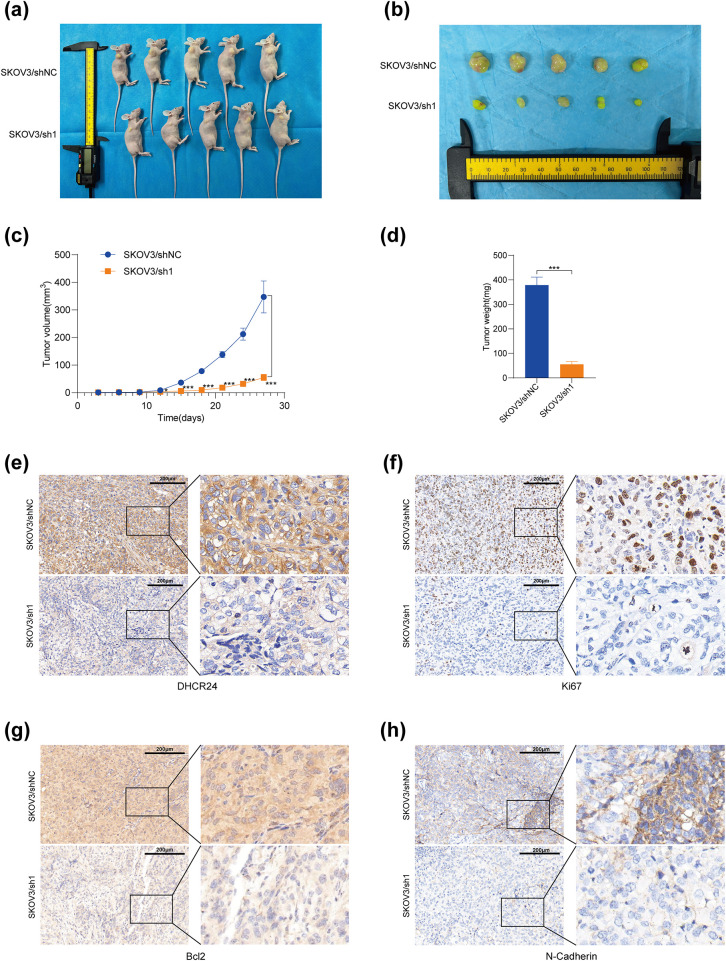
DHCR24 knockdown inhibits tumor growth *in vivo*. (**a**) OC tumors in mice in the SKOV3/shNC and SKOV3/sh1 groups; (**b**) Subcutaneous tumors in the SKOV3/shNC and SKOV3/sh1 groups; (**c**) Growth curves of subcutaneous tumors in the SKOV3/shNC and SKOV3/sh1 groups; (**d**) Weights of subcutaneous tumors in the SKOV3/shNC and SKOV3/sh1 groups; (**e–h**) DHCR24, Ki67, Bcl2 and N-cadherin expression levels in subcutaneous tumors of the SKOV3/shNC and SKOV3/sh1 groups, detected using IHC. Note: DHCR24, 3β-hydroxysterol 24-reductase; IHC, immunohistochemical. *p < 0.05, ***p < 0.001

Mice were randomly categorized into the SKOV3/sh1 (DHCR24 low expression), SKOV3/shNC (negative control), A2780/DHCR24 (DHCR24 overexpression), and A2780/Vector (control) groups. The tumor volume, tumor growth rate, and weight decreased (Fig. 5a–d) in the DHCR24 low-expression group. Therefore, we inferred that DHCR24 knockdown inhibits OC cell proliferation *in vivo*. IHC indicated that DHCR24, Ki67, Bcl2, and N-cadherin expression levels were higher in the SKOV3/shNC group than in the SKOV3/sh1 group (Fig. 5e–h). Additionally, IHC results further showed higher expression of TGF-β, P-Smad2, and P-Smad3 in the SKOV3/shNC group, while E-cadherin expression exhibited the opposite pattern (Fig. S4). Furthermore, tumor volume, tumor growth rate, and weight increased significantly in the DHCR24 overexpression group, suggesting that DHCR24 overexpression promotes OC cell proliferation *in vivo* (Fig. S5). The IHC results were consistent with those of the SKOV3 group (All *p* < 0.01) (Fig. S6).

### 3.8 DHCR24 Promotes the TGF-***β***1 Pathway in OC Cells

**Figure 6:**
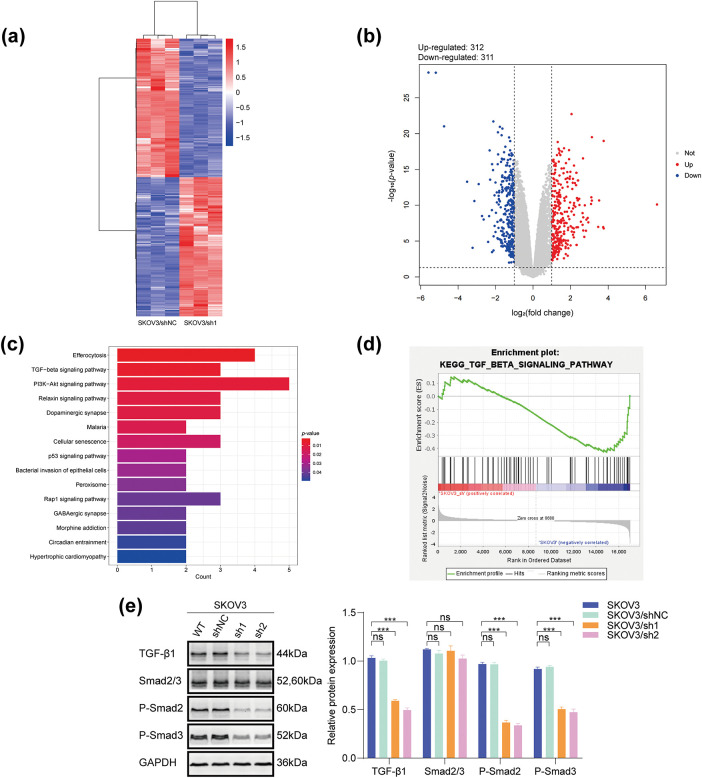

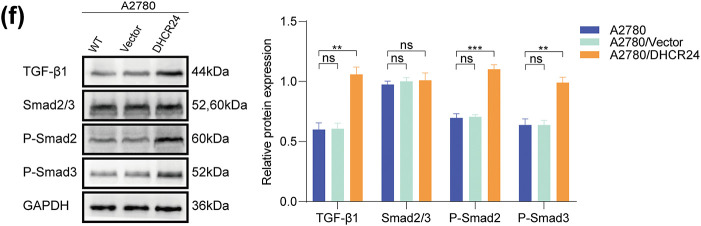
DHCR24 correlates with the TGF-β1 pathway in OC cells. (**a**) Heat map of differentially expressed genes in SKOV3/shNC and SKOV3/sh1 cells (red indicates highly expressed genes, blue indicates lowly expressed genes, *p*-value < 0.05); (**b**) Volcanic map of differentially expressed genes in SKOV3/shNC and SKOV3/sh1 cells (|logFC| > 1 and adjusted *p*-value < 0.05); (**c**) KEGG pathway enrichment analysis reveals a significant correlation between DHCR24 and the TGF-β signaling pathway; (**d**) GSEA shows that DHCR24 might act on ovarian cancer through the TGF-β pathway; (**e**) Left: Changes in key proteins of the TGF-β signaling pathway before and after DHCR24 knockdown, detected using WB. Right: Corresponding quantitative analysis results. The experiment was independently repeated three times; (**f**) Left: Changes in key proteins of the TGF-β signaling pathway before and after DHCR24 overexpression, detected using WB. Right: Corresponding quantitative analysis results. The experiment was independently repeated three times. Note: GEPIA, Gene Expression Profiling Interactive Analysis; KEGG, Kyoto Encyclopedia of Genes and Genomes; GSEA, Gene Set Enrichment Analysis; DHCR24, 3β-hydroxysterol Δ24-reductase; TGF-β1, Transforming growth factor beta 1. ns, no significance; **p < 0.01, ***p < 0.001

SKOV3/sh1 and SKOV3/shNC cells were utilized for transcriptome sequencing. Heat maps and volcano plots showed differentially expressed genes between SKOV3/sh1 and SKOV3/shNC cells (Fig. 6a,b). Data analyses suggested a significant correlation between DHCR24 and the TGF-β pathway in OC (Fig. 6c,d). Subsequently, we detected key protein molecules of the TGF-β pathway based on WB results. In SKOV3 cells, TGF-β1 and p-Smads expression levels were lower in the DHCR24 low-expression group (SKOV3/sh1, sh2) than those in the control group (SKOV3/shNC, SKOV3); however, Smad2/3 levels remained unchanged (Fig. 6e). The reverse was also true in A2780 cells (All *p* < 0.01) (Fig. 6f).

### 3.9 The OC Malignancy-Promoting Effect of DHCR24 Correlates with the TGF-**β**1 Pathway

**Figure 7:**
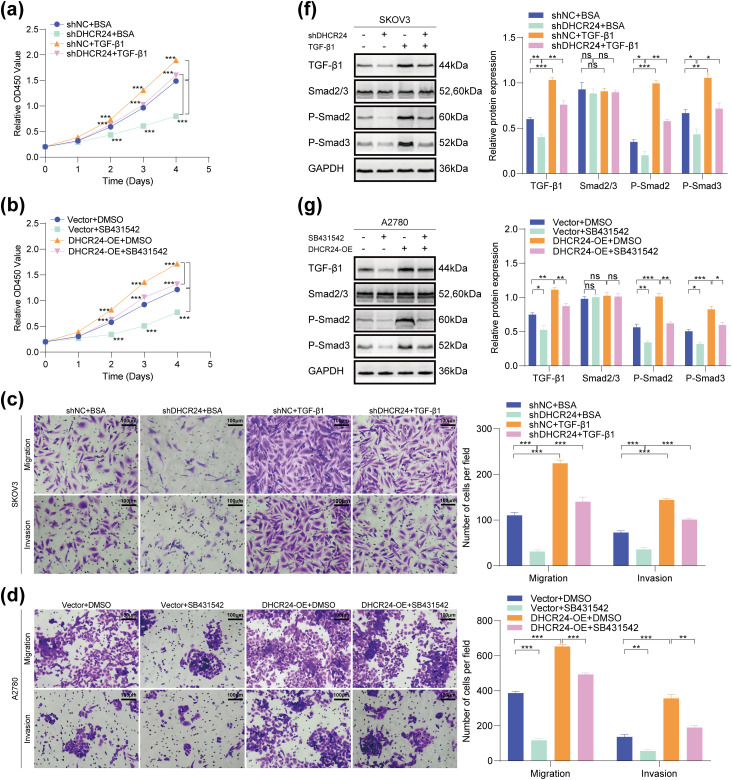

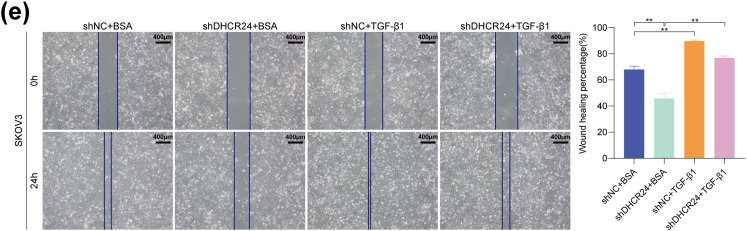
The OC malignancy-promoting effect of DHCR24 correlates with the TGF-β pathway. (**a**) Effects of treatment with or without TGF-β1 on the proliferation capacity of SKOV3 cells, determined using the CCK-8 assay (3 × 10^3^ cells/well); (**b**) Effects of treatment with or without SB431542 on the proliferation capacity of A2780 cells, determined using the CCK-8 assay (3 × 10^3^ cells/well); (**c**) Effects of treatment with or without TGF-β1 on the migration and invasion ability of SKOV3 cells, determined using the Transwell assay. Right: Corresponding quantitative analysis results (2 × 10^4^ cells/well); (**d**) Effect of treatment with or without SB431542 on the migration and invasion ability of A2780 cells, determined using the Transwell assay. Right: Corresponding quantitative analysis results (6 × 10^4^ cells/well); (**e**) Effect of treatment with or without TGF-β1 on the migratory ability of SKOV3 cells, determined using the wound healing assay. Right: Corresponding quantitative analysis results; (**f**) Effect of treatment with or without TGF-β1 on key protein molecules involved in the TGF-β pathway in SKOV3 cells, determined using WB. Right: Corresponding quantitative analysis results; (**g**) Effect of treatment with or without SB431542 on key protein molecules involved in the TGF-β pathway in A2780 cells, determined using WB. Right: Corresponding quantitative analysis results. All experiments were independently repeated three times. Note: CCK-8, cell counting kit-8; DHCR24, 3β-hydroxysterol 24-reductase; TGF-β1, Transforming growth factor beta 1. ns, no significance; *p < 0.05, **p < 0.01, ***p < 0.001

In SKOV3 cells, DHCR24 knockdown reduced their malignant behavior, whereas TGF-β1 treatment reversed this effect (Fig. 7a,c,e). In A2780 cells, DHCR24 overexpression enhanced their malignant behavior, but SB431542 treatment reversed this effect (Fig. 7b,d). In addition, WB results indicated that DHCR24 knockdown decreased the levels of key proteins of the TGF-β1 pathway in SKOV3 cells, whereas TGF-β1 treatment reversed this effect (Fig. 7f). In A2780 cells, DHCR24 overexpression increased the levels of key proteins of the TGF-β1 pathway, whereas SB431542 treatment reversed this effect (all *p* < 0.05) (Fig. 7g). Collectively, these findings indicated that DHCR24 regulates OC cell malignant behavior and correlates with the TGF-β1 pathway.

### 3.10 SH-42 Suppressed the Proliferation, Migration, and Invasion Capabilities of OC Cells

**Figure 8:**
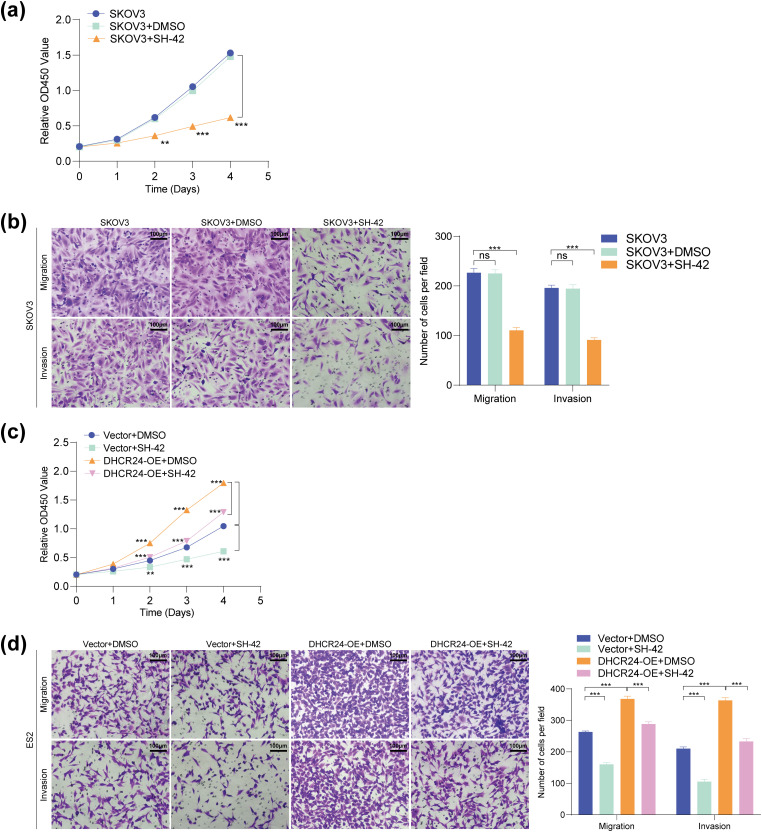
SH-42 suppressed the proliferative, migration, and invasion capabilities of OC cells. (**a**) CCK-8 assay showing the effect of SH-42 on the proliferative ability of SKOV3 cells (3 × 10^3^ cells/well); (**b**) Transwell assay showing the effect of SH-42 on the migration and invasion of SKOV3 cells (2 × 10^4^ cells/well); (**c**) CCK-8 assay showing the effects of DHCR24 overexpression and SH-42 on the proliferation of ES2 cells (3 × 10^3^ cells/well); (**d**) Transwell assay showing the effects of DHCR24 overexpression and SH-42 on the migration and invasion of ES2 cells (2 × 10^4^ cells/well). All experiments were independently repeated three times. Note: CCK-8, cell counting kit-8; DHCR24, 3β-hydroxysterol Δ24-reductase; ns, no significance; **p < 0.01, ***p < 0.001

To investigate whether DHCR24 can serve as a potential therapeutic target for OC, SKOV3, and ES2 cells were treated with the DHCR24 inhibitor SH-42. Results showed that SH-42 reduced the proliferation, migration, and invasion capabilities of SKOV3 (Fig. 8a,b) and ES2 cells (Fig. 8c,d). Further, SH-42 also reversed the malignancy-promoting effects of DHCR24 overexpression in ES2 cells (Fig. 8c,d). In summary, SH-42 can significantly reduce the cancer-promoting effects of DHCR24 in OC cells.

## 4 Discussion

OC has the highest mortality rate among gynecologic malignancies [1]. Therefore, further research is essential to elucidate the mechanisms of OC development for early diagnosis and improved treatment. The present findings demonstrated that DHCR24 expression is upregulated in OC and promotes malignant behavior and EMT in OC cells. In addition, DHCR24 was found to act on OC via the TGF-β pathway. Based on our results, we inferred that DHCR24 is positively correlated with TGF-β1 and p-Smads levels. Notably, TGF-β1 reversed the inhibitory effect of DHCR24 knockdown on the malignant behavior of OC cells, while SB431542 reversed the promoting effect of DHCR24 overexpression on these processes. Additionally, experiments involving treatment with the DHCR24 inhibitor SH-42 suggested that it could reverse the malignancy-promoting effects of DHCR24 in OC cells. In summary, our findings demonstrated that DHCR24 significantly promoted OC cell invasion and metastasis by activating the TGF-β signaling pathway. Clinical data revealed that high DHCR24 expression in OC tissues was significantly associated with poor patient prognosis, and its regulation of the TGF-β pathway represented a key mechanism underlying chemotherapy resistance [35,36]. These results suggested that targeting DHCR24 might represent a promising strategy for OC precision therapy, particularly when combined with TGF-β pathway inhibitors, showing potential clinical significance.

The role of DHCR24 has been well-characterized in various cancers. In cervical precancerous lesions, hucMSC-sEV inhibits cell migration by targeting DHCR24 through miR-370-3p [37]. A study also reveals that DHCR24 participates in the generation and progression of tumor cells by regulating intracellular reactive oxygen species [12]. Moreover, DHCR24 can regulate tumor cell apoptosis by activating the PI3K-AKT signaling pathway, a process that may be mediated by Ras [38]. However, its specific function in OC has not been fully elucidated. DHCR24 promotes cholesterol synthesis, providing raw materials for cancer cell membrane formation and signal transduction, thereby facilitating proliferation [39]. In this study, DHCR24 knockdown suppressed OC cell proliferation, which may be associated with decreased cholesterol levels. Additionally, DHCR24 acts as a ROS scavenger with antioxidant functions, protecting cancer cells from radiotherapy/chemotherapy-induced DNA damage while inhibiting apoptosis [7,22]. The significant increase in OC cell apoptosis following DHCR24 knockdown in this study may be related to its antioxidant role. Existing studies suggest that cholesterol synthesis promotes the aggregation of TβRII in lipid rafts, enhancing its sensitivity to TGF-β ligands [40–42]. DHCR24 may facilitate TβRII accumulation in lipid rafts and activate the TGF-β signaling pathway by promoting cholesterol synthesis. In summary, DHCR24 likely promotes malignant progression through multidimensional mechanisms (metabolic reprogramming, antioxidant effects, and anti-apoptosis). Further experiments are needed to explore these mechanisms in depth.

**Figure 9:**
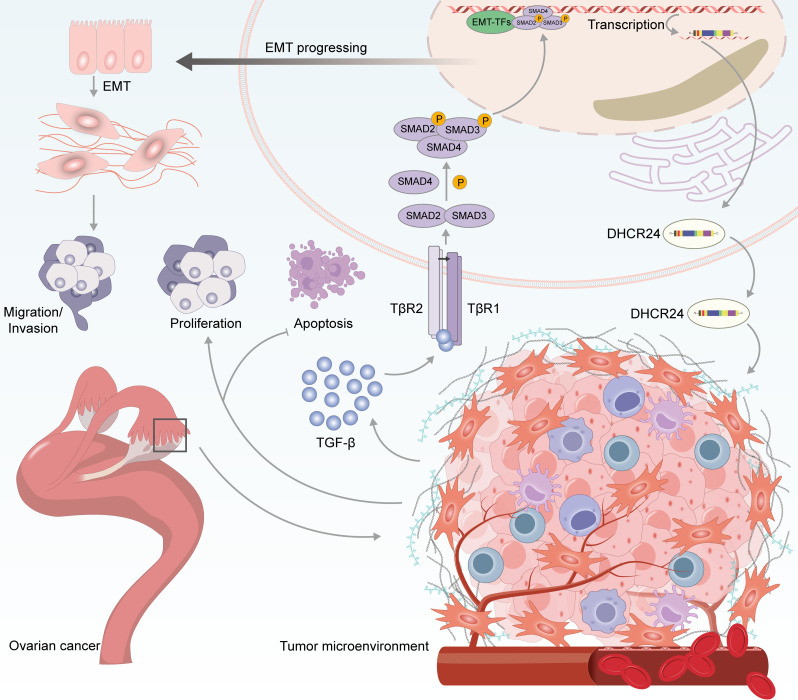
Schematic diagram showing the proposed role of DHCR24 in promoting cancer via modulating the TGF-β1 pathway

TGF-β is an essential predisposing factor in the EMT process and may promote cancer progression via multiple mechanisms [43–45]. In OC, TGF-β1 plays a vital role in the maintenance of the cancer stem cell phenotype, and tumor metastasis [46–48]. In this study, we found that DHCR24 correlates with the EMT of OC cells and increases the levels of key proteins involved in the TGF-β1 pathway. To explore the effect of DHCR24 on the TGF-β1 pathway, we treated SKOV3 and A2780 cells with TGF-β1 and SB431542, respectively. Our research suggests that DHCR24 regulates OC cell malignant behavior via the TGF-β1 pathway. Our findings preliminarily summarize the role of DHCR24 in promoting cancer by regulating the TGF-β1 pathway, as illustrated in Fig. 9.

Previous studies have reported that DHCR24 promotes tumor progression by regulating cholesterol metabolism in hepatocellular carcinoma [39] and diffuse large B-cell lymphoma [49]. However, our study revealed that DHCR24 drives tumor migration and invasion through TGF-β signaling pathway activation, thereby expanding its experimental basis in oncology. These findings also provide a theoretical foundation for developing OC-specific DHCR24 inhibitors (e.g., in combination with TGF-β inhibitors). As this study only preliminarily explored DHCR24’s regulation of the TGF-β pathway, and given the extensive crosstalk between TGF-β1 signaling and pathways such as PI3K/AKT [50–52], future studies should further validate the specific molecular mechanisms through mass spectrometry, proteomics, and co-immunoprecipitation experiments. While multiple studies suggest DHCR24 as a potential therapeutic target in cancer [8,16,53], our findings demonstrate that the small-molecule inhibitor SH-42 can suppress OC cell malignancy, though its application may be limited by off-target effects and drug resistance induction [54], necessitating further investigation of its safety and efficacy.

Although TGF-β1 inhibitors have shown potential in preclinical studies, their clinical translation still faces several challenges. Because of the extensive cross-talk between the TGF-β1 pathway and other signaling pathways, TGF-β1 inhibitors may activate other pro-tumor signaling pathways, thereby reducing their therapeutic efficacy [50]. Furthermore, tumor cell resistance to TGF-β1 inhibitors poses another significant challenge, as stromal cells in the tumor microenvironment may counteract the effects of TGF-β1 inhibitors by secreting paracrine factors [55]. Therefore, issues related to selectivity, off-target risks, and drug resistance remain critical challenges to be addressed.

This study has certain limitations. As the availability of clinical samples and pathological data is limited, future studies will aim to expand the clinical cohort, increase the sample size in mouse experiments, and collect additional pathological data to strengthen the findings on the correlation between DHCR24 expression and OC prognosis, while enhancing the credibility of our research conclusions. Furthermore, studies incorporating *in vivo* models that better mimic the complexity of human biochemistry may provide more clinically relevant insights into DHCR24’s potential as a therapeutic target in OC.

## 5 Conclusions

The OC malignancy-promoting effect of DHCR24 correlates with the TGF-β pathway. Our findings suggest that DHCR24 facilitates the development of OC, and its inhibition is likely to be a therapeutic strategy for OC.

## Supplementary Materials



## Data Availability

The data that support the findings of this study are openly available in the Sequence Read Archive at https://www.ncbi.nlm.nih.gov/bioproject/PRJNA1206547 (accessed on 14 May 2025) or are available from the Corresponding Author, Liangdan Tang, upon reasonable request.

## Supplementary Materials

The supplementary material is available online at https://www.techscience.com/doi/10.32604/or.2025.065451/s1.

## Glossary

ANOVA: Analysis of Variance
BSA: Bovine serum albumin
CCK-8: Cell counting kit-8
DHCR24: 3β-hydroxysterol Δ24-reductase
DMSO: Dimethyl sulfoxide
EMT: Epithelial–mesenchymal transition
EOC: Epithelial ovarian cancer
FIGO: International Federation of Gynecology and Obstetrics
GEPIA: Gene Expression Profiling Interactive Analysis
GSEA: Gene Set Enrichment Analysis
IACUC: Institutional Animal Care and Use Committee
IHC: Immunohistochemistry
KEGG: Kyoto Encyclopedia of Genes and Genomes
OC: Ovarian cancer
PBS: Phosphate-buffered saline
PFS: Progression-free survival
qPCR: Quantitative PCR
TCGA: The Cancer Genome Atlas
TGF-β: Transforming growth factor beta
WB: Western blot
