# Supplementary material for: 3β-Hydroxysterol Δ24-Reductase Promotes Ovarian Cancer Progression by Activating the TGF-β1/Smad2/3 Signaling Pathway
Source: Oncol Res. 2025 Sep 26;33(10):3041–64. doi: 10.32604/or.2025.065451 (PMC12494117; doi:10.32604/or.2025.065451)
Supplement: Supplementary file 1 [file OncolRes-33-65451-s001.docx]

**Table S1:** Relationship between DHCR24 expression and the clinicopathological parameters of ovarian cancer.

| **Characteristics** | | **Cases (%)** | **DHCR24 expression** | | ***p*-value** |
| --- | --- | --- | --- | --- | --- |
|  |  |  | **High expression n=48 (%)** | **Low expression n=22 (%)** |  |
| **Age (years)** | <55 | 24 (34.3) | 14 (29.2) | 10 (45.5) | 0.183 |
|  | ≥55 | 46 (65.7) | 34 (70.8) | 12 (54.5) |  |
| **Pathological**  **type** | SOC | 51 (72.9) | 38 (79.2) | 13 (59.1) | 0.080 |
|  | Other* | 19 (27.1) | 10 (20.8) | 9 (40.9) |  |
| **FIGO Stage** | I & II | 16 (22.9) | 7 (14.6) | 9 (40.9) | 0.015 |
|  | III & IV | 54 (77.1) | 41 (85.4) | 13 (59.1) |  |
| **CA125** | <1000 U/mL | 41 (58.6) | 26 (54.2) | 15 (68.2) | 0.269 |
|  | ≥1000 U/mL | 29 (41.4) | 22 (45.8) | 7 (31.8) |  |
| **Lymphatic**  **metastasis** | NO | 32 (45.7) | 20 (41.7) | 12 (54.5) | 0.315 |
|  | YES | 38 (54.3) | 28 (58.3) | 10 (45.5) |  |
| **Ascites** | NO | 31 (44.3) | 17 (35.4) | 14 (63.6) | 0.027 |
|  | YES | 39 (55.7) | 31 (64.6) | 8 (36.4) |  |
| **Tumor size** | <5 cm | 29 (41.4) | 18 (37.5) | 11 (50.0) | 0.324 |
|  | ≥5 cm | 41 (58.6) | 30 (62.5) | 11 (50.0) |  |

Note: FIGO, International Federation of Gynecology and Obstetrics; SOC, serous ovarian cancer. *Clear cell carcinoma, endometrioid carcinoma, mucinous carcinoma.

**Table S2:** Primer sequences used in this study for quantitative PCR.

| **Primer** | **Forward** | **Reverse** |
| --- | --- | --- |
| **DHCR24** | 5’-TGAAGACAAACCGAGAGGGC-3’ | 5’- CAGCCAAAGAGGTAGCGGAA-3’ |
| **GAPDH** | 5’-AAATCCCATCACCATCTTCCAG-3’ | 5’-ATGAGTCCTTCCACGATACCAA-3’ |

**Table S3:** Antibodies used in this study for western blotting.

| **Antibodies** | **Product No. and Source** | **Dilution** |
| --- | --- | --- |
| DHCR24 Antibody（rabbit monoclonal） | #2033, Cell Signaling Technology, MA, USA | 1:1000 |
| E-Cadherin Antibody（rabbit monoclonal） | #3195, Cell Signaling Technology, MA, USA | 1:1000 |
| N-Cadherin Antibody（rabbit monoclonal） | #13116, Cell Signaling Technology, MA, USA | 1:1000 |
| Vimentin Antibody（rabbit monoclonal） | #5741, Cell Signaling Technology, MA, USA | 1:5000 |
| Bax Antibody（rabbit monoclonal） | #5023, Cell Signaling Technology, MA, USA | 1:1000 |
| Bcl2 Antibody（rabbit monoclonal） | #3498, Cell Signaling Technology, MA, USA | 1:1000 |
| TGF beta 1 Antibody（rabbit monoclonal） | ab215715, Abcam, Cambridge, UK | 1:1000 |
| Smad2/3 Antibody（rabbit monoclonal） | #8685, Cell Signaling Technology, MA, USA | 1:1000 |
| Phospho-Smad2 (Ser465/467) Antibody（rabbit monoclonal） | #3108, Cell Signaling Technology, MA, USA | 1:1000 |
| Phospho-Smad3 (Ser423/425) Antibody（rabbit monoclonal） | ab52903, Abcam, Cambridge, UK | 1:2000 |
| HRP Conjugated AffiniPure Goat Anti-rabbit IgG (H+L) | BA1054, BOSTER Biological Technology Co. Ltd, Wuhan, China | 1:10000 |
| HRP Conjugated AffiniPure Goat Anti-mouse IgG (H+L) | BA1050, BOSTER Biological Technology Co., Ltd, Wuhan, China | 1:10000 |
| β-Actin （rabbit polyclonal） | 380624, ZenBioScience, Chengdu, China | 1:5000 |
| GAPDH （rabbit monoclonal） | 380626, ZenBioScience, Chengdu, China | 1:5000 |

**Table S4:** shRNA sequences used in this study.

| **shRNA** | **Sense** | **Antisense** |
| --- | --- | --- |
| **Negative control** | 5’-UUCUCCGAACGUGUCACGUTT-3’ | 5’-ACGUGACACGUUCGGAGAATT-3’ |
| **DHCR24-shRNA-1** | 5’-CUACGUGUCGGGAAGUACA-3’ | 5’-UGUACUUCCCGACACGUAG-3’ |
| **DHCR24-shRNA-2** | 5’-CGCUUAUCUUCGAUAUCUA-3’ | 5’-UAGAUAUCGAAGAUAAGCG-3’ |
| **DHCR24-shRNA-3** | 5’-ACAUCGACAUUGGAGCAUA-3’ | 5’-UAUGCUCCAAUGUCGAUGU-3’ |

**Table S5:** Antibodies used in this study for immunohistochemical staining.

| **Antibodies** | **Product No. and Source** | **Dilution** |
| --- | --- | --- |
| DHCR24 Antibody （rabbit polyclonal） | #2033, Cell Signaling Technology, MA, USA | 1:100 |
| Ki67 Antibody (mouse monoclonal)  Bcl2 antibody (rabbit monoclonal)  TGF beta 1 Antibody（rabbit monoclonal）  Phospho-Smad2 (Ser467) （rabbit monoclonal）  Phospho-Smad3 (Ser423/425) Antibody（rabbit monoclonal）  E-Cadherin Antibody（rabbit monoclonal）  N-Cadherin Antibody（rabbit monoclonal） | GB121141-50, Servicebio, Wuhan, China  R22494, ZenBioScience, Chengdu, China  ab215715, Abcam, Cambridge, UK  ab28088, Abcam, Cambridge, UK  ab52903, Abcam, Cambridge, UK  #3195, Cell Signaling Technology, MA, USA  #13116, Cell Signaling Technology, MA, USA | 1:500  1:100  1:500  1:2000  1:200  1:400  1:200 |

**Figure S1:** DHCR24 promotes OC cell proliferation. (a) Transfection efficiency of DHCR24-shRNA in CAOV3 cells, detected using qPCR. (b) Left: WB results confirming the establishment of an *in* *vitro* model with stable low DHCR24 expression. Right: Corresponding quantitative analysis. (c) Transfection efficiency of DHCR24 overexpression in A2780 cells, detected using qPCR. (d) Left: Establishment of an *in* *vitro* model with stable DHCR24 overexpression, verified using WB. Right: Corresponding quantitative analysis. (e) CCK-8 assay showing that DHCR24 overexpression promotes OC cell proliferation. (3×10^3^ cells/well). (f) Left: Colony formation assay showing that DHCR24 overexpression promotes OC cell proliferation. Right: Corresponding quantitative analysis (4×10^2^ cells/well). (g) CCK-8 assay showing that DHCR24 knockdown inhibits OC cell proliferation (3×10^3^ cells/well). (h) Left: Colony formation assay showing that DHCR24 knockdown inhibits OC cell proliferation. Right: Corresponding quantitative analysis (4×10^2^ cells/well). All experiments were independently repeated three times. Note: DHCR24, 3β-hydroxysterol 24-reductase; OC, ovarian cancer; qPCR, quantitative polymerase chain reaction; WB, western blot; CCK-8, cell counting kit-8. ns, no significance; **p*<0.05, ***p*<0.01, ****p*<0.001.

**Figure S2:** DHCR24 inhibits apoptosis of OC cells. (a). Left: Apoptosis of OC cells before and after DHCR24 overexpression, detected using flow cytometry. Right: Corresponding quantitative analysis. (b). Left: Apoptosis of OC cells before and after DHCR24 knockdown, detected using flow cytometry. Right: Corresponding quantitative analysis. (c). Left: Changes in apoptosis-related proteins Bax and Bcl2 before and after DHCR24 overexpression, detected using WB. Right: Corresponding quantitative analysis. (d). Left: Changes in apoptosis-related proteins Bax and Bcl2 before and after DHCR24 knockdown, detected using WB. Right: Corresponding quantitative analysis. All experiments were independently repeated three times. Note: DHCR24, 3β-hydroxysterol Δ24-reductase; OC, ovarian cancer; WB, western blot. ns, no significance; ***p*<0.01, ****p*<0.001.

**Figure S3:** DHCR24 promotes migration, invasion, and EMT of OC cells. (a) Left: Transwell assay results showing that DHCR24 knockdown inhibits OC cell migration and invasion. Right: Corresponding quantitative analysis (CAOV3: 6 × 10^4^ cells/well). (b) Left: Transwell assay results showing that DHCR24 overexpression promotes OC cell migration and invasion. Right: Corresponding quantitative analysis (A2780: 6 × 10^4^ cells/well). (c) Left: Wound healing experiment results showing that DHCR24 knockdown inhibits the migration of OC cells. Right: Corresponding quantitative analysis results. (d) Left: Changes in EMT-related proteins in OC cells before and after DHCR24 knockdown, detected using WB. Right: Corresponding quantitative analysis. (e) Left: Changes in EMT-related proteins in OC cells before and after DHCR24 overexpression, detected using WB. Right: Corresponding quantitative analysis. All experiments were independently repeated three times. Note: DHCR24, 3β-hydroxysterol Δ24-reductase; OC, ovarian cancer; EMT, epithelial-mesenchymal transition; WB, western blot. ns, so significance; ***p*<0.01, ****p*<0.001.

**Figure S4:** Knockdown of DHCR24 inhibits tumor growth *in* *vivo*. (a-d) TGF-β1, P-Smad2, P-Smad3, and E-cadherin expression levels in subcutaneous tumors of the SKOV3/shNC and SKOV3/sh1 groups, detected using IHC. Note: DHCR24, 3β-hydroxysterol Δ24-reductase; IHC, immunohistochemistry.

**Figure S5:** DHCR24 overexpression promotes tumor growth *in* *vivo*. (a) OC tumors in mice in the A2780/DHCR24 and A2780/Vector groups. (b) Subcutaneous tumors in the A2780/DHCR24 and A2780/Vector groups. (c) Growth curves of subcutaneous tumors in the A2780/DHCR24 and A2780/Vector groups. (d) Weights of subcutaneous tumors in the A2780/DHCR24 and A2780/Vector groups. (e-h) DHCR24, Ki67, Bcl2, and N-cadherin expression levels in subcutaneous tumors of the A2780/DHCR24 and A2780/Vector groups, detected using IHC. Note: DHCR24, 3β-hydroxysterol Δ24-reductase; IHC, immunohistochemistry. ****p*<0.001.

**Figure S6:** DHCR24 overexpression promotes tumor growth *in* *vivo*. (a-d) TGF-β1, P-Smad2, P-Smad3, and E-cadherin expression levels in subcutaneous tumors of the A2780/DHCR24 and A2780/Vector groups, detected using IHC. Note: DHCR24, 3β-hydroxysterol Δ24-reductase; IHC, immunohistochemistry.
